# 24-hour blood pressure homeostasis and renal function in subjects with and without metabolic syndrome

**DOI:** 10.1186/1758-5996-7-S1-A38

**Published:** 2015-11-11

**Authors:** Bárbara Limberger Nedel, Leticia Maria Tedesco Silva, Monique de Moura Machado, Rodrigo Soares de Souza Marques, Leonardo de Andrade Mesquita, Luciana Pavan Antoniolli, Tassia Cividanes Pazinato, Vanessa Piccoli, Mayara Abichequer Beer, Anize Delfino von Frankenberg, Fernando Gerchman

**Affiliations:** 1 Universidade Federal do Rio Grande do Sul, Porto Alegre, Brazil

## Background

Metabolic syndrome (MS) is related with progressive decrease of renal function. Although hypertension has a major role on this relationship, it is not clear how its behavior is related to decreased renal function.

## Objective

To study how blood pressure (BP) measured by 24-hour ambulatory BP is related to renal function in patients with and without MS.

## Materials and methods

We designed a cross-sectional study of consecutive individuals (n=108; females 74%; 52.8±12.7 yrs.; mean±SD) from the Diabetes Clinic of a university hospital. MS was defined by the International Diabetes Federation criteria. BP was measured at office and its circadian variation was determined by 24-h ambulatory BP monitoring, after withdrawal from anti-hypertensive medications. Patients were classified according to their BP behavior: normotension (NT; n=29), white-coat hypertension (WCH; n=19) and ambulatory hypertension (AHT; n=57). Fasting and 2h-plasma glucose levels, lipid profile, creatinine and 24-h urinary albumin excretion (UAER) were measured. Glomerular filtration rate (GFR) was estimated by the CKD-EPI equation. A two-sided P value <0.05 was considered significant.

## Results

Estimated GFR (EGFR) was lower in subjects with MS than in those without MS (Mean±SD; 90±20 vs 98.8±16.5; P=0.047). EGFR was related to age (r=-0.666; P<0.001), fasting glucose (r=0.223; P=0.021), and 24-h systolic BP (r=-0.196; P=0.044), but not to diastolic BP. EGFR was inversely related to sleep-time BP (r=-0.224; P=0.021), morning systolic BP (r=-0.224; P=0.030) and pulse pressure (r=-0.233; P=0.170). Subjects with WCH and AHT compared to those with NT had lower EGFR (Mean±SD; 89.3±18 vs 89.6±26.3 vs 100.2±14.8; P=0.036) and higher UAER (Median [P25-75]; 1 [0-5.3] vs 6.1 [1-19] vs 6.3 [1-16.8]; P=0.031).

## Conclusion

A higher sleep-time BP, morning systolic BP, and pulse pressure were the components of BP homeostasis mostly related to decreased renal function and may be taken into account in the assessment of subjects with MS. The findings should prompt further research in order to evaluate whether or not interventions impacting on BP during sleep-time and early morning period may prevent renal damage.

